# ¿Somos iguales? Using a structural violence framework to understand gender and health inequities from an intersectional perspective in the Peruvian Amazon

**DOI:** 10.1080/16549716.2017.1330458

**Published:** 2017-06-22

**Authors:** Geordan D. Shannon, Angelica Motta, Carlos F. Cáceres, Jolene Skordis-Worrall, Diana Bowie, Audrey Prost

**Affiliations:** ^a^ Institute for Global Health, University College London (UCL), London, UK; ^b^ DB Peru, Lima, Peru; ^c^ Centro de Investigación Interdisciplinaria en Sexualidad, Sida y Sociedad, Universidad Peruana Cayetano Heredia (UPCH), Lima, Peru

**Keywords:** Gender and Health Inequality - intersections with other relevant axes of oppression, Gender, social determinants of health, intersectionality, structural violence, Amazon

## Abstract

**Background**: In the Peruvian Amazon, historical events of colonization and political marginalization intersect with identities of ethnicity, class and geography in the construction of gender and health inequities. Gender-based inequalities can manifest in poor health outcomes via discriminatory practices, healthcare system imbalances, inequities in health research, and differential exposures and vulnerabilities to diseases. Structural violence is a comprehensive framework to explain the mechanisms by which social forces such as poverty, racism and gender inequity become embodied as individual experiences and health outcomes, and thus may be a useful tool in structuring an intersectional analysis of gender and health inequities in Amazonian Peru.

**Objective**: The aim of this paper is to explore the intersection of gender inequities with other social inequalities in the production of health and disease in Peru’s Amazon using a structural violence approach.

**Design**: Exploratory qualitative research was performed in two Loreto settings – urban Iquitos and the rural Lower Napo River region – between March and November 2015. This included participant observation with prolonged stays in the community, 46 semi-structured individual interviews and three group discussions. Thematic analysis was performed to identify emerging themes related to gender inequalities in health and healthcare and how these intersect with layered social disadvantages in the reproduction of health and illness. We employed a structural violence approach to construct an intersectional analysis of gender and health inequities in Amazonian Peru.

**Results**: Our findings were arranged into five interrelated domains within a gender, structural violence and health model: gender as a symbolic institution, systemic gender-based violence, interpersonal violence, the social determinants of health, and other health outcomes. Each domain represents one aspect of the complex associations between gender, gender inequity and health. Through this model, we were able to explore: gender, health and intersectionality; structural violence; and to highlight particular local gender and health dynamics. Intersecting influences of poverty, ethnicity, geography and gender served as significant barriers to healthcare in both rural and urban settings.

## Background

In the Peruvian Amazon, historical events linked to colonization and political marginalization intersect with identities of ethnicity, class and geography in the construction of gender and health inequities. Peruvian society is built on a hierarchy of power, economic, ethnic and gender relations [1–3]. Thus, the analysis of gender and health inequities must be positioned within a discussion of the broader, societal-level influences that impede the full realization of individual and community rights and wellbeing.

Peru’s three geopolitical regions are symbolically organized into a hierarchy [1,2,4]. The coastal desert represents the most modern region, home to the capital Lima and the concentration of political and economic power [2]. The sierra region is associated with Andean culture and positioned as the source of grand pre-Hispanic civilizations [1,4]. The jungle region, despite covering over 60% of the Peruvian land mass, does not have an important presence in national dynamics and is neglected in the national psyche or positioned as distant and marginalized [1]. Discourses arising from colonization portrayed the Amazon as ‘frontier territory,’ a virgin space of abundance home to primitive communities [1]. The perception of virgin frontier territory has been invoked to justify exploitation of natural resources, and, in turn, shaped the construction of gender roles and sexuality in this region [1,5]. It is in this context that gender dynamics are constructed and in turn affect health outcomes in Peru’s Amazon.

Localized gender roles and inequities, interpreted in the context of broader societal inequities, may explain health risks, behaviours and outcomes. For example, economic inequality in Peru – reflected by a Gini Coefficient of 45.3 (down from 56.4 in 1999) and an Inequality-Adjusted Human Development Index of 0.563 – correlates with inequities in reproductive, maternal and child health outcomes [6,7]. In the context of geographic remoteness coupled with widespread poverty in the Amazon region, transactional and commercial sex work performed by largely marginalized *ribereños  *(Ribereño refers to a person who resides in a rural (normally agricultural) river community. This is similar to the term campesino, a term referring topeasant farmers in mountainous parts of Peru) has been shown to drive risks of sexually transmitted disease and HIV transmission [8]. Furthermore, rural Peru has some of the world’s highest reported rates of gender-based violence (GBV): the World Health Organization (WHO) Multi-Country Study on Women’s Health and Violence Against Women identified a lifetime prevalence of 61% [9]. Gender inequities may manifest in poor health outcomes via discriminatory practices, inequitable health service provision, inequities in health research, and differential exposures and vulnerabilities to diseases [10]. However, this does not occur in isolation from other harmful social and structural forces.

Structural violence is a comprehensive framework to explain the mechanisms through which social forces such as poverty, racism and gender inequity become embodied as individual experiences and health outcomes [11–14]. The term ‘structural violence’ refers to institutionalized social structures, such as poverty, racism and gender inequity, that prevent people from meeting their basic needs [11,15]. A structural violence framework recognizes and critiques intersecting inequities in the suppression of human potential and may be a useful tool in structuring an intersectional analysis of gender and health inequities in Amazonian Peru.

The aim of this paper is to explore the intersection of gender inequities with other social inequalities in the production of health and disease in Peru’s Amazon. We use a structural violence framework to guide an analysis of qualitative interviews and field research on gender, health and intersectionality in Peru’s Loreto region.

## Design

### Local context

The department of Loreto in the north east of Peru is covered by Amazonian floodplains. Research occurred in two locations: the port city of Iquitos and the rural communities of the Lower Napo River (LNR). This allowed for a more nuanced exploration of social and structural factors relevant to both urban and rural settings.

Loreto’s capital, Iquitos, is one of the world’s most remote and inaccessible cities. Iquitos can only be accessed via air or water, as it is situated in the heart of the Amazon jungle. It is a busy port city of over 400,000 inhabitants, many of whom are originally from surrounding jungle communities [16]. Iquitos is distinctly jungle-flavoured: the humid streets are buzzing with local moto-taxi transport, regularly tropical Latino music blares from bars and cafes, and the Belen marketplace teems with wild animals, jungle meats, traditional medicine and local produce. Closer to the centre of town, low-level concrete dwellings predominate, whereas those who can’t afford this live in shanty wooden structures towards the city peripheries. Iquitos has a number of primary healthcare posts and three main hospitals. Of these, Hospital Regional (HR) caters for a usually poor population who are on social and health benefits (*Seguro Integral de Salud, SIS*).

Around 12 hours upstream by riverboat are the communities of the Lower Napo, a group of 25 villages comprising approximately 5000 inhabitants in total. Each community consists of about 200 people who live a predominantly subsistence agricultural life. Normally, the community is built around a central football field, a concrete primary school building and a *maloca* (an open-air meeting hut). Families construct their houses from wood and clear land for small-scale agriculture involving chickens and pigs. Health services are sparse. Each community elects a health *promotor* (community health worker) and a *partera* (lay midwife). There are three health posts in the 25 communities staffed by a health *técnico* (a health worker with higher education). The main referral post is around four hours by boat upstream. Although they have Indigenous heritage, many identify as *mestizo  *(Mixed-race, referring to an individual’s Indigenous heritage) *ribereños*. The river and the jungle are significant influences in people’s lives; this is reflected in local cultural practices.

### Community access and institutional approval

Research was facilitated through the organization DB Peru (www.dbperu.org) a non-govermental organization (NGO) based in the LNR that partners with local communities to provide access to health knowledge and services. GDS first started working as a medical doctor with DB Peru in 2014, and spent nine months undertaking immersive fieldwork in addition to medical outreach work in both Iquitos and the communities of the LNR in 2015. Researchers collaborated with the Universidad Peruana Cayetano Heredia (UPCH) Centre for Interdisciplinary Studies in Sexuality, AIDS and Society in Lima. Ethical approval was secured from both UCL (Project ID: 5406.001) and UPCH (Codigo SIDISI: 63685).

### Study design

The main element of the qualitative research was a series of semi-structured individual interviews and group discussions. The topic guides covered four broad domains: understandings of gender equality (GE) and local gender dynamics; factors influencing gender roles and inequalities; the connection between gender and health; and questions around individual empowerment. Each domain contained a series of questions and probes to further explore areas of interest. These questions were derived from the Encuesta Demográfica y de Salud Familiar (ENDES) household survey and informed by current literature on gender equality and empowerment. The interview questions were drafted in April 2014, and refined following consultation with experts in London and Peru in May 2014.

Field notes were recorded in video, audio, photographic and written forms. Much fieldwork was performed in settings without electricity; therefore, some notes were handwritten. During the fieldwork itself, initial notes were very instructive or descriptive. During periods of rest and reflection, notes were more structured around interactions with individuals and the community. Field notes and video were used as adjunct information to support the thematic analysis of the transcripts, detailed below. In an iterative manner, notes and video were recorded to reflect on emerging themes, ideas and challenges of the research process.

### Data collection and analysis

We recruited patients through local health services. In Iquitos, women were approached in the clinic outpatient waiting area in a semi-targeted, semi-opportunistic manner to ensure a balanced selection of age and backgrounds (Table 1). In the LNR, women were approached via a *promotor* (a lay community health worker). Both verbal and written consent were necessary due to anticipated low literacy rates. Naturally forming groups of service providers were selected to represent a range of community services (Table 2). A team of three research assistants led by GDS performed the interviews in Spanish (Castellano).

**Table 1.** Characteristics of individual participants, by geographic location.

Location	LNR (n = 22)	Iquitos (n = 21)
Mean age (range), years	44 (19–70)	34 (16–53)
Professional backgrounds	Predominantly agricultural, some community members had dual (voluntary) roles as health *promotores* or Programa Juntos leaders	Teachers, healthcare staff, housewives, mothers and government professionals
Gender breakdown	22 women	21 women

**Table 2.** Overview of group discussions.

Group details	Overview of group
Health *promotores* (lay health workers) (n = 19)	Health *promotores* from up to 25 communities come together for health education with DB Peru annually in a small town, Mazan. Sixteen men and three women participated in April 2015. On the last day of education, GDS gathered all participants in the community *maloca* and spent four hours discussing their roles, their opinion on gender dynamics and other gender-specific health issues in the community.
Primary and secondary school teachers (n = 8)	Mangua is one of the larger communities of the LNR, and eight teachers live here semi-permanently to run the primary and secondary schools. During an afternoon meeting, GDS sat down with all of them (five women and three men) and discussed key gender issues they have observed.
Volunteer staff at charity (n = 3)	GDS spent two hours interviewing staff (one woman, two men) from a local Iquitos charity, the Arco Iris Orphanage, who had insight into not only children/family issues, but also the local Iquitos community dynamics.

A professional transcription service in Lima provided Spanish-language transcripts. GDS and AP analysed transcripts in the primary language (Spanish) with an English code applied to selected blocks of text, using NVivo 11.0 for Windows and Mac. Quotes were selected to demonstrate particular emerging themes, and at this point were translated into English. AM and CFC checked the translation.

An initial open, inductive coding approach was used, and these codes were subsequently refined into emerging themes. Thematic analysis is a flexible methodological approach that reports, analyses and interprets patterns within data, beyond explicit words or phrases, to identify emerging ideas and themes [17–19]. It can be used to find solutions to real-world problems by building upwards from local information [19] and may be a useful approach to the identification of local gender and social inequalities. These themes were first evaluated in relation to the geographic location. Overarching themes relevant to both locations were analysed together. Themes derived from the data were then organized using a structural violence framework, operationalized as described next, to identify and explain intersections of gender and other social categories such as ethnicity and poverty.

### Research framework: operationalizing structural violence

We employ a structural violence framework to construct an intersectional analysis of gender and health inequities in Amazonian Peru.

Structural violence is a comprehensive framework to explain the mechanisms through which social forces such as poverty, racism and gender inequity become embodied as individual experiences and health outcomes [11]. First introduced by Galtung and Latin American liberation theologians in the 1960s, structural violence refers to institutionalized social structures, such as poverty, racism and gender inequity, that prevent people from meeting their basic needs [11,15]. Galtung defined violence as ‘the cause of the difference between the potential and the actual, between what could have been and what is’ [20, p. 168]; thus violence refers broadly to harm caused to individuals and is positioned as ‘…the avoidable impairment of fundamental human needs’ [15,21]. However, this violence is indirect, as it functions through the replication of ubiquitous, normalized social structures [11].

**Figure 1.**
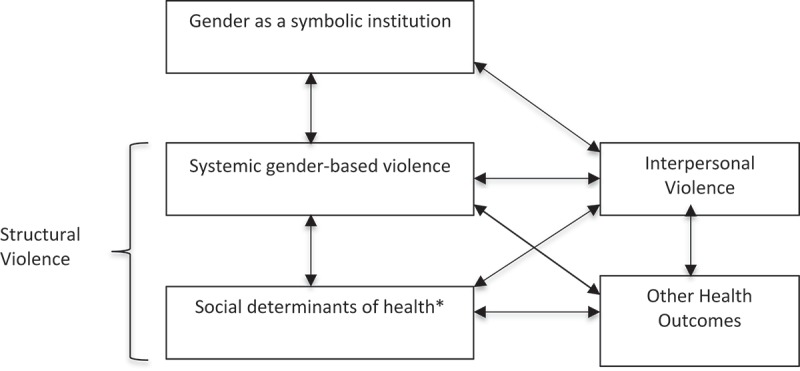
The relationship between symbolic, systemic and individual violence, and how these relate to gender and health outcomes, as derived from Montesanti and Thurston (2015) [23]. *Social determinants of health as identified by Montesanti and Thurston (2015) [23] include: social support, personal health practices, income and social status, education, child development, employment and working conditions, social environments, culture, welfare institutions, civil society, economic institutions.

Advocates such as Farmer have used the concept of structural violence to understand and address health inequities [11–14]. Through this lens, modern communicable epidemics such as HIV/AIDS and tuberculosis are positioned as inseparable from, and a result of, ‘violent’ socioeconomic structures [12,13]. More recently, Montesanti and Thurston overlaid the concept of structural violence onto an ecological framework for health [22] by mapping the role of symbolic, structural, and interpersonal violence on women’s lives [23]. Through this process, intersecting social determinants of health are linked both to structural violence and to individual health outcomes, including interpersonal violence (Figure 1) [23].

### Structural violence and intersectionality

We used the conceptual diagram detailed in Figure 1 to structure our results into five interrelated domains within a gender, structural violence and health model: gender as a symbolic institution, systemic gender-based violence, interpersonal violence, the social determinants of health, and other health outcomes. This allowed for an analysis of the intersections of gender inequities with other social inequalities in the production of health and disease in Peru’s Amazon.

Intersectionality is a research paradigm that attempts to understand how ‘race, class, gender, sexuality, ethnicity, nation, ability, and age operate not as unitary, mutually exclusive entities, but rather as reciprocally constructing phenomena’ [24, p. 2]. First coined by African American feminist scholars, intersectionality ‘moves beyond single or typically favoured categories of analysis (e.g. sex, gender, race and class) to consider simultaneous interactions between different aspects of social identity… as well as the impact of systems and processes of oppression and domination’ [25, p. 2]. As such, it emphasizes that human lives cannot be reduced to a single social or physical characteristic, that certain characteristics are socially constructed and fluid, and that human experiences cannot be understood by prioritizing a single characteristic over another nor can they be conceptualized as additive binary factors [26].

When exploring the relationship between gender, structural violence and health, an intersectionality approach positions gender in a broader social, historical and political context and thus allows for the consideration of intersecting influences of poverty, geography, age and indigeneity in the construction of health and violence in the Amazon. The structural violence framework also allows the simultaneous consideration of multiple levels of influence, from the individual through to the environment, and of how these act in complex, overlapping ways.

## Results

Individual interview participants included 21 women from Iquitos, and 22 women from the LNR. Participants’ ages ranged from 18 to 71 years of age, with a spectrum of educational, professional and life backgrounds (Tables 1 and 2). Three group interviews comprised a group of 19 health *promotores* from various communities of the LNR, a group of eight teachers on placement in the LNR and the board of directors of an orphanage in Iquitos.

### Gender as a symbolic institution

#### Gender equality

In river communities, gender equality was conceptualized as equality of opportunity by some: ‘Both men and women are equal before society. They have the same opportunities’ (C, female interviewee, LNR). Others saw gender equality as mutual respect between men and women: ‘Well [in] my personal opinion, gender equality is understood as mutual respect, both the woman and the man, I understand its limitations of course, it is basically mutual respect’ (FT, female FGD, LNR). Furthermore, gender equality was believed to be about the freedom to express opinions and feelings:

…gender equality could also be seen from a point where all people are equal, only what sets us apart is the difference in gender between the man and woman. But we all think, are all free to express what we feel. (MT, male FGD, LNR)

In Iquitos, there was a divide between the ideal and the lived reality of GE:

I have read and heard on the radio that we all now have equal rights, both men and women, and there is no discrimination, the law says, but in reality it is not achieved, because many places discriminate against women, or for older people who no longer have a job, are not able to work, it’s not achieved, the law says so but equality is not met. (L, female interviewee, Iquitos)

However, some city-dwellers recognized the dynamic nature of gender roles and how gender equality could be achieved through transforming both men and women’s roles:

For me gender equality is that men and women are able to have the same rights and responsibilities, not only with respect to work but for example, in society the old mothers raised their children, to say you know what my child, you are not able to do such a thing in the house because the wife is in charge. Then the woman also gets tired, the woman has the right to enjoy her free time. So, what happens? Men normally do certain things, certain occupations, women no. Therefore, for me gender equality is that the man can be a help to his wife, he can work the same way as his wife, develop the children in an equal way to his wife. (MC, female interviewee, Iquitos)

#### Gender roles and stereotypes

*Machismo* – an exaggerated form of masculinity – and *marianismo* – virtuous femininity – emerged as examples of deep-rooted, local gender stereotypes and behaviours in Iquitos. Although machismo was explicitly identified in many interviews, marianismo was instead implied through discussions around how women were expected to behave in relation to *machista* men:

Men of Loreto are very macho […] they don’t let women explain themselves, study, have their own money, they want the women to depend on them. And they do not share their things, for them the woman is only for washing, cooking and attending the children… Maybe not all, very few men value women. And I know people from the north… they are more affectionate, more loving, more chivalrous… As a woman from Iquitos, I am proud to be here, I just do not like the custom of men here. (O, female interviewee, Iquitos)

As an emic concept, the term machismo was mainly used to imply sexism or sexist behaviour, as opposed to a more broadly accepted etic discourse of hyper-masculinity. At a societal level, machismo was linked to systematic factors, such as unequal access to education, under- or un-employment, and the overall social environment:

The majority are studying yes, because they find work, when they are professionals they change [from being violent], but a person who has not studied, that has only completed fifth grade of secondary school, has very few possibilities to find a good place of work to progress economically and socially. (D, female interviewee, Iquitos)

Whilst *ribereño* communities were in general perceived as being more peaceful and egalitarian, there was a stark difference in gender roles between men and women. For example, observers from the LNR report that many women in comparison to their male counterparts lacked voice or agency:

What we see here is that women do not express what they feel, what they think… gender equality is not being fulfilled here, we are meeting men and women, and everyone expresses their opinion, their point of view. But in a meeting, the women sit to the side. They are separate, silent and the men are the ones who have opinions… (MT, male FGD, LNR)

This lack of voice was further accentuated when *ribereños* came to Iquitos to seek healthcare:

Mostly when the people come from the periphery, they come, as a couple, both men and women, and the person who informs us about the patient is a man, not the woman, the one with a cell phone is a man, not a woman, and who has the money is a man, not the woman. Yes, I have seen, on many occasions I have seen. Sometimes one says, sir, I’m not asking you, I want her to tell me. No, it is that she does not know how to respond. That is the answer. It is that she doesn’t know how to respond. (A, female FGD, Iquitos)

Although this was interpreted as a relationship power differential, it may also be explained by lower levels of education and self-confidence, or by a culture where it is a tradition to let the male partner speak. Not only did many rural dwellers have low levels of health literacy and low self-esteem, women were further disadvantaged by their Indigenous heritage or poverty: ‘There are girls who come from the river… they feel inhibited by their poverty, by their features too. Sometimes they may feel less than a city girl, and this doesn’t necessarily have to be’ (T, female interviewee, Iquitos). Thus, structural inequities enact violence upon the individual through the internalization of shame or stigma about one’s cultural, geographic or economic position.

### Systemic gender-based violence

That’s a problem that exists every day, the only thing I can say is that every day there is violence, violence, violence, violence. (T, female interviewee, Iquitos)

Violence can be traced through Peruvian history, from the invasion and colonization of the native, predominantly Incan, society by Spanish conquistadors in the 1530s, to a military dictatorship (and subsequent violent resistance movements such as the *Sendero Luminoso*) throughout the second half of the twentieth century [2,24 27]. Through the 1990s the authoritarian government led by Fujimori appropriated popular feminist discourse around family planning to push a ‘forced sterilization’ surgical contraception programme targeted mainly at rural and Indigenous women [24 27]. A history of systematic violence is reflected by current rates of GBV in rural areas: data demonstrate a national lifetime prevalence in rural Peru of up to 61% [25 28]. Recent figures released from Loreto by the organization Promsex show that 79% of women between the ages of 18 and 29 in Mazan, a small town located between Iquitos and the communities of the Napo River, had experienced sexual violence at some point in their life [26 29].

It is in this context that violence against women (VAW) is an accepted, widespread cultural phenomenon in Iquitos, often linked to machismo behaviour:

Because this city’s society is machista, men usually denigrate women, women alone are responsible for what is inside the house. They can’t find work, they suffer from domestic abuse, and this does not let the women develop as a person, as an individual, so I think women are not on the same level as men here. (MC, female interviewee, Iquitos)

These societal attitudes reflect systemic violence by reinforcing female inferiority and weakness, and perpetuating and normalizing domestic violence (DV). These factors subsequently impeded women’s personal growth, wellbeing and physical integrity.

Although violence was widely discussed in Iquitos, in the rural LNR it was widely known but under-reported and largely remained ‘hidden.’ *Ribereño* communities consist of around 200 people: a small-town dynamic operated whereby many appeared to be aware of those who perpetrated or suffered from violence, yet would be reticent to discuss it openly. Furthermore, individual lack of voice and disempowerment were seen as barriers to addressing violence in the community:

They don’t report [DV]. Here’s the governor and lieutenant governor, police, municipal agent. But no, they do not report their husbands, for fear and dread. They live in their houses, the man goes to look for food and fish and she is quiet. Perhaps by lack of knowledge or ignorance, perhaps if she is struck she does not denounce those things, in all that they are. (FT, female interviewee, LNR)

Alternative explanations to under- or non-reporting may be a lack of knowledge about how to report an incidence, not feeling it is a public issue and thus being ashamed to share openly, and not perceiving the situation as violent secondary to the normalization of violence in the community and/or household.

Within the family unit, violence may be enacted to compensate for powerlessness in the face of widespread structural violence:

There are some families who have no resources to get ahead, youth who are forced to work and don’t study… For example, if you start work from the 3rd grade and down the street, there will be crime, violence, prostitution, drunkenness. (GM, female interviewee, Iquitos)

Violent behaviour was recognized as an intergenerationally transmitted cycle:

When a child grows up watching his father abandon his mother, or she is beaten, maltreated, then that creates in a child a psychological disorder. When this person is an adult they will do the same with her husband and their children. So it is a given string. (MC, female interviewee, Iquitos)

At the same time, being a witness to their father’s violent behaviour against their mother may reinforce the marianista element of men’s cultural background, and possibly the idea of their mother as a saint or a martyr.

At a broader structural level, participants recognized systemic discrimination against women that impeded leadership opportunities:

There are not many [women politicians]. We don’t have many opportunities… Because they say that the woman does not govern well, say the men, this is why they don’t vote for women. For example, here in Iquitos, only one time has a female governed as mayor, from here no one more, one time here in the time we have lived here. There are not more women… because there is discrimination happening to women, they don’t give us opportunities so that a woman would govern. According to people, they don’t have the ability, they don’t give women opportunities. (L, female interviewee, Iquitos)

Female leaders were seen as potential role models to empower women to speak up and prevent violence:

Realistically, the people are perhaps missing something, a female leader, someone from the community who gets up in front and says, you know what, no more abuse of women, no more, perhaps it takes a decision on behalf of the women. Or perhaps they do not know what rights they also have as women. Apart from other rights they also have, for lack of education. (M, female interviewee, LNR)

### Interpersonal violence

There are so many victims of beatings, abuse, mistreatment, every day in Iquitos. (J, female interviewee, Iquitos)

Many women shared explicit stories of DV perpetrated against either themselves or their neighbours:

Yes I have seen a neighbour who suffers from this. Every time I came her husband beat her like this so drunk. She had to run, hiding in my house she lives, she runs every time she sees her husband, to hide, to sleep in another house with the children. (E, female interviewee, Iquitos)

This was often related to spousal alcohol use – perhaps a reflection of internalized self-violence through alcohol which then facilitated externalized violence towards others: ‘because sometimes they have physical abuse from their spouse and that too when their husbands are drunk at times lack food for their children, this is a problem also’ (G, female interviewee, LNR). Significant disruption to women and children’s lives was associated with leaving situations of violence, such as lacking money for food or needing to sleep in others’ houses: ‘When you depend economically on men, you cannot leave. And that’s why sometimes they endure violence’ (R, female interviewee, Iquitos).

When asked about the specific impact of violence on health, interviewees articulated how family violence affects women not only physically, but also emotionally. Women spoke freely of the psychological impact of violence on women, a theme that links aspects of violence, behaviour and individual self-esteem:

Yes for sure, how many women have been here in the hospital that come with bruises, cuts. So, that’s physical damage, but mostly the damage may be a greater extent, I think psychological, because the psychological damage is not easily erased. (MC, female interviewee, Iquitos)

Another outcome of DV, and perhaps a factor in individual vulnerability and resilience against it, is women’s self-esteem:

What may help is to have respect for women. It is having enough self-esteem, because as a woman who lets you stay and hit her again and again and stays the same, it is that she has nowhere to go. Or because she has no economy, because she depends on the husband… As she lets it continue happening, it will always happen. And that has to do with self-esteem, women must be valued, be respected, and know that it is equal for all, both men and women… (T, female interviewee, Iquitos)

### Social determinants of health

How does one heal if there is no money, here is the problem when there is no money. (N, female interviewee, LNR)

In the impoverished population of Loreto, money was seen as power towards health: ‘Without money in hospital you are not valued at all. If you don’t have family, no one helps you’ (R, female interviewee, LNR). Many spoke of how, as a poor, rural woman, you were not only neglected in the system, but also experienced active discrimination from healthcare staff. Subtler forms of discrimination included how the system was structured to be inaccessible to those from more rural areas who had poor literacy and healthcare knowledge:

For example, if you see a person who comes from the river, from the farms, as they are called, and don’t have someone to orient you, nobody heeds and no one teaches you, no one says, you’re going there, there you have to touch in, they do not know, no one looks at her, it is a type of discrimination to people of the rivers, the poor women, it is one type of discrimination in my opinion. (T, female interviewee, Iquitos)

Social inequalities were more difficult to discuss in rural river communities. Women’s life perspective was often more limited, and remained uninfluenced by external media or opinion. Many women conceptualized their life in relation to others who were in a similar position to them in their community. Some had heard of discrimination and/or issues around Indigenous cultures, and many had experienced discrimination whilst in Iquitos. They, however, did not seem to identify discrimination if and when it occurred; perhaps this was due to a sense of fatalism in that *ribereños* were always going to remain powerless, an internalized sense of shame, or a lack of language with which to discuss these issues. Many women’s dialogue in river communities focused around equality with others and a sense of community very localized to the geographic area.

Concepts of health and disease in Amazonian societies have been recognized as holistic and family oriented [30]. Health is conceptualized as not merely the absence of illness but overall wellbeing through healthy relationships and self-esteem [30]. It is in this context that interviewees positioned the relationship between gender equality and health as symbiotic: ‘Because health is almost equal to women’s lives, if you don’t care for yourself, you are sick. And equal also in women, if you’re not good, if you’re not in your peaceful house, feeding your children, it can be bad’ (C, female interviewee, Iquitos). This statement not only highlighted the holistic Amazonian life view but also reinforced the pervasive nature of structural violence in the production of women’s health: the ubiquitous nature of gender and social disparities meant that health and gender were perceived as being intimately connected.

### Other health outcomes: reproductive health

Despite family planning services falling under the auspices of publicly available government health services, three overarching structural issues combined to perpetuate a form of structural violence that impeded healthcare access.

First, family planning clinics were overburdened with staff shortages, resource shortages and overall stress on the system. Excessive waiting times, particularly for non-urgent and preventative healthcare, were strong disincentives:

Well here the girls, because they are pregnant early in age, many young women who are pregnant and this, I don’t know what this is from lacking, from advice, or parents, because at an early age they are already pregnant… Because even for family planning you need to get an appointment. And that should not be. (R, female interviewee, Iquitos)

Some women reported their first choice of contraceptive was often not available in local clinics that they depend on. Within a pressurized system driven on a tight budget, staff dissatisfaction ran high: on the one hand, many service providers reportedly were short or rude towards patients, and on the other hand staff themselves were often striking to protest unfair wages and work conditions. Violence is passed down through the system to harm patients, the least powerful consumer of healthcare.

Second, sexual and reproductive healthcare (SRH) services were not perceived as sensitive to the needs of the population. In regional areas such as the LNR where a lottery system of service provision placements to underserved rural areas occurred, mainly male service providers occupied positions of administering contraception and family planning education in remote communities. This operated in discordance to many women’s desires of discussing intimate relationship details with a female provider. Conversely, in the higher-resourced Iquitos city, the majority of SRH was provided by *obstetrices* (nurse midwives), an overwhelmingly female-dominant service. In this situation, female service providers discussed ‘women’s business’ – by women, for women. This placed the burden of contraception onto the woman, reinforced stereotypes of women being reproductive objects and excluded men from important discourses around sexuality and reproduction.

Third, government-run sterilization programmes of the 1990s reportedly violated human rights and targeted marginalized (rural, poor, ethnic minority) women, including participating communities [24]. One interviewee reportedly thought she was having a pap smear when she was sterilized without consent; she was incorrectly told that officials were just ‘…taking her uterus out and cleaning it’ (W, female interviewee, LNR). This programme had a significant impact on women’s health, serving to reinforce an individual lack of power and health knowledge, and has implications for the trust women place in their interactions with government SRH services in this region [24].

## Discussion

The aim of this paper was to explore gender inequalities in health and how they intersect with other social inequalities in Amazonian Peru using a structural violence approach. Our findings were arranged into five interrelated domains within a gender, structural violence and health model: gender as a symbolic institution, systemic gender-based violence, interpersonal violence, the social determinants of health, and other health outcomes. Each domain represents one aspect of the complex associations between gender, gender inequity and health. Through this model, we were able to explore: gender, health and intersectionality; structural violence; and to highlight particular local gender and health dynamics.

### Intersectionality, gender and health

Basing interviews in both rural and urban health systems of Loreto provided a privileged insight into individual experiences of intersecting aspects of social oppression and disempowerment. In addition to gender, intersecting layers of poverty, geography and ethnicity impacted individuals’ experience of the healthcare system. Women from rural river communities were perceived to be the most disadvantaged when interacting with the healthcare system, weighed down by layered gender inequity, poverty, geographic remoteness, and stigma relating to ethnicity. This appeared to translate into a loss of voice or, worse still, invisibility within the overall system.

The healthcare system was a mirror to reflect more widespread social inequities. In a society such as Peru that is built on social inequality, the healthcare system perpetuated a similar social hierarchy. Inequity in this regard was driven most overtly by economic status, where individual value and the quality of treatment received from staff were perceived to be influenced by how much money you had. Despite setting the research in hospitals providing services for those on the SIS, a social-insurance healthcare programme ensuring coverage for the most disadvantaged, a certain power differential was replicated through interactions between the system, staff and individual patients. This may reflect the apparent resource scarcity in Loreto, and how, in the context of an overburdened system, social inequities are replicated and exaggerated. It also reflects how structural violence is conferred through the system onto the patients who are most economically vulnerable.

The association between gender, gender inequity and health is complex. Not only does gender intersect with other social identities in the production of health at an individual level, but also gender – as it relates to an individual and their health – must be positioned within a broader understanding of household, community, environmental and societal factors. The structural violence model, as utilized in this article, is sensitive to intersecting identities at an individual level whilst simultaneously recognizing surrounding multi-level social and structural determinants.

### Use of a structural violence model

In the context of the Peruvian Amazon, a structural violence approach was able to identify facilitators of social wellbeing (or conversely, oppression) that act to influence individual health and disease outcomes. As a conceptual framework that is sensitive to various social and structural influences on health, it allows consideration and discussion of intersecting factors including gender, poverty, geography and ethnicity. Furthermore, it facilitates reflection on the inextricable link between historical events and current inequities, something that is pertinent to the discussion of culture and health in the Amazon31. As a tool that recognizes the complex links between an individual and their environment and positions the individual at the centre of multi-level health determinants, it functions in complement to the ecological framework for health [22].

Distilling such complex phenomena and interactions into a single model risks oversimplification or misrepresentation of the gender and health interface. The structural violence approach, as applied by Farmer towards health inequity, has been criticized as being blunt to the full spectrum of human inequality by conflating ‘…full-fledged domination with mere social disparity … [collapsing] forms of violence that need to be differentiated such as physical, economic, political, and symbolic variants or those wielded by state, market, and other social entities’ [31]. Furthermore, evidence supporting the structural violence research paradigm is often in the form of case studies that place inequities as being out of the reach of individuals. Whilst this is often the case, this risks furthering individual powerlessness by assuming that solutions to inequities are located outside the individual or community. Our approach on the one hand enabled a description of intersecting social factors in the relationship between gender and health, but, on the other, was unable to facilitate action on these inequities.

### Reflections on gender, health and sexuality in the Amazon

This research was based in two relatively representative locations in the Peruvian Amazon: the port city of Iquitos and the rural communities of the LNR. Whilst both settings have distinct gender and health dynamics, there was naturally a lot of overlap between the two due to increasing human mobility and urban migration, especially in the younger generation. In river communities, health was particularly influenced by traditional ways that were gender-segregated. This observation is supported by Espinoza’s exploration of gender and ethnic spirituality in Amazonian communities, where women were positioned as vulnerable to certain diseases and were affected by taboos on bodily functions such as menstruation yet simultaneously expected to fulfil primary caregiver roles in facilitating family healthcare [31]. In urban contexts, gender and health behaviours were more removed from traditional ways and more open to broader societal and media influences. For example, hegemonic masculinity may influence male health-seeking behaviours and restrict access to healthcare.

The concepts of gender, equality and health were intimately linked through a holistic life view of health and disease prominent in Amazonian communities. This extends to the interrelatedness of humans and nature. It is argued that the exploitation of natural resources is analogous to the construction of women’s roles and sexuality in this region [1]. Amazonian women have been described as having an excessively eroticized sexuality, and meeting male sexual demands liberally and wildly, summarized by the colloquial term ‘charapa ardiente’ (burning charapa^1^ ^1^
*Charapa* is a term to refer to someone who was born in the jungle.) [5]. The ‘charapa ardiente’ may also function symbolically as a personification of what a ‘decent’ woman should not be and thus define by contrast ideal feminine behaviour, such as more traditional constructs of virginal or virtuous femininity derived from Catholicism. This dichotomy of emptiness/virginity and abundance/sexuality is replicated around both natural resources and female stereotypes. Male roles are also influenced by a history of natural resource exploitation: men of this region hold a hardworking service mentality, born out of poverty and influenced by a history of colonization. Local constructs of machismo, sexuality and violence are shaped by the natural environment, traditional spirituality and economic oppression [31].

### Gender, violence and health

Oppression is a result of many conditions, not the least of which reside in the consciousness. [12]

In the face of overwhelming systemic disadvantage, both women and men in different ways internalized this oppression, subsequently expressing limited aspirations and identity. In women, this manifested in lack of voice, timid behaviour and ‘feeling less’ when seeking healthcare. In men, the internalization of systemic violence often led to violence directed against the self and others, and may serve to explain the high rates of interpersonal violence in this region. Such gender differences seemed to result from different demands on men and on women based on a gender structure, but such demands were not necessarily enforced by the partner of the opposite gender at the family level. They were also imposed by structural factors including institutions (namely, health and education), economic and employment opportunities and cultural norms. It is through a structural violence lens that hierarchies, power, sexism, classism, racism and capitalism may be used in the understanding of individual experiences of violence.

The act of violence, it has been argued, is a reflection of many things. Although DV represents a single incident of an individual acting out sexual or physical power over another, it may also reflect the violent milieu of a particular society, from the physical environment to harmful cultural norms. Just as the exploitation of natural resources in the Amazon is analogous to the sexual exploitation of women, human relationships with the natural environment have been described as ‘rape’ [30]. Centuries of colonization and resource extraction in the Amazon can be seen as violence against nature, this ‘rape’ forcing a further disconnection not only from the land but also valuable social and cultural fabric intrinsic to Amazonian life. Although interpersonal violence cannot completely be explained by societal or historical events, the structural violence framework positions individual acts of violence within a broader context, capturing the complexity of the interplay between multiple spheres of influence.

Violence may represent the violence of society being focused through an individual man onto an individual woman [32]. Alternatively, aggression may manifest as an action to compensate for individual perceptions of social powerlessness [32]. A discussion around human oppression is particularly relevant to understanding violence perpetuated via Loretano men. Loreto is an economically poor province, particularly in rural areas, where many face a daily struggle for survival. As Kaufman argues:

The daily work life of industrial, class societies is one of violence… It is violence that condemns the majority to work to exhaustion for forty or fifty years and then to be thrown into society’s garbage bin for the old and used-up. The racism, sexism, and heterosexism that have been institutionalized in our societies are socially regulated acts of violence. [32]

‘Industry,’ as it relates to rural Loreto, includes the physically arduous agricultural labour undertaken in *ribereño* communities living in entrenched conditions of poverty.

## Conclusion

This study sought to explore gender, health and intersectionality in the Loreto region of Peru using a structural violence lens. The construction of gender is intimately linked to the history of colonization and environmental exploitation in this region. Concepts of emptiness/virginity, abundance/sexuality and oppression/marginalization through intersecting forces of structural violence combine to influence gender roles, sexuality, interpersonal relations, and, ultimately, health.

Beyond providing a unique insight into local gender dynamics of the Peruvian Amazon, we were able to dissect layered social and structural influences on health through the lens of structural violence. Overlapping and cumulative influences of poverty, ethnicity, geography and gender served as significant barriers to both GE and healthcare in both rural and urban settings.

## Paper context

Whilst intersectionality and structural violence frameworks have both separately been used to explore either health or violence against women, to our knowledge this is the first attempt to combine the two approaches into an analysis of the intersections of gender, violence and health. Beyond providing a unique insight into local gender dynamics of the Peruvian Amazon, we were able to dissect layered social and structural influences on health through the lens of structural violence.
